# Supplementary material for: Patient-perceived progression in multiple system atrophy: natural history of quality of life
Source: J Neurol Neurosurg Psychiatry. 2024 Mar 7;95(9):804–11. doi: 10.1136/jnnp-2023-332733 (PMC11347237; doi:10.1136/jnnp-2023-332733)
Supplement: Supplementary data [file jnnp-2023-332733supp001.pdf]

Supplementary materials

Patient-perceived progression in multiple system atrophy: natural history of quality of life
Saulnier et al., *Journal of Neurology, Neurosurgery and Psychiatry*

Figure S1: Flowchart of MSA sample selection.

Patients without any completed MSA-QoL questionnaire, with missing values for at least one covariate of interest (among sex, age, subtype, diagnosis certainty, presence or absence of orthostatic hypotension at inclusion, presence or absence of urinary disorder at inclusion, delay since symptom onset, and treatments), and without at least one item completed per (modified) dimension were excluded from the analyses.

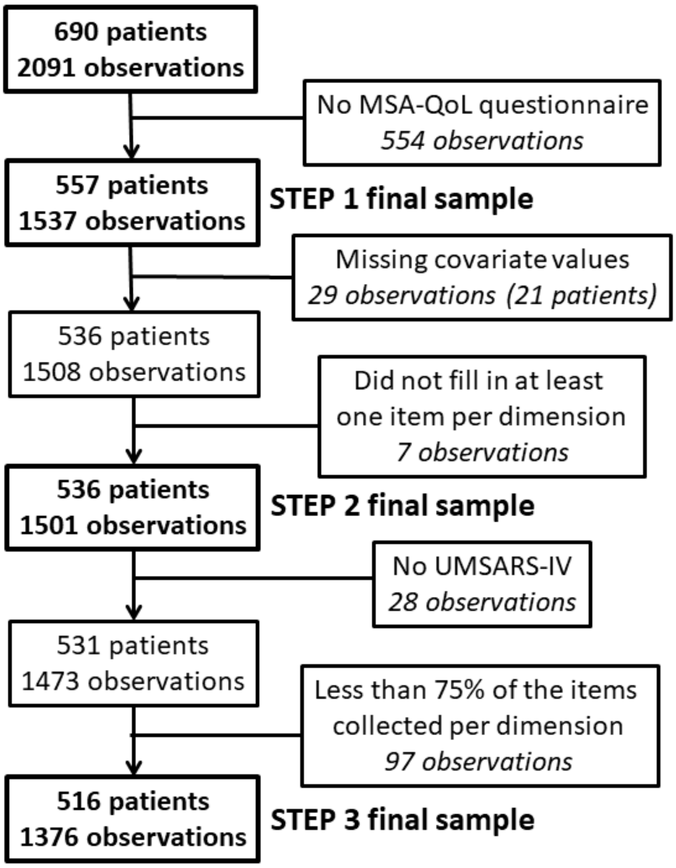

Footnote: **STEP 1** corresponds to the identification of the scale subdimensions; **STEP 2** corresponds to the description of each subdimension trajectory over time; **STEP 3** corresponds to the mapping of subdimension items with the disease stages.

Saulnier T, *et al. J Neurol Neurosurg Psychiatry* 2024; 95:804–811. doi: 10.1136/jnnp-2023-332733

Figure S2: Mean trajectories of Hr-QoL items over time predicted by a joint longitudinal IRT model for each modified-dimension.

Trajectories are represented for the reference profile: a male patient, diagnosed with probable MSA-P, aged 65 years old at inclusion, with orthostatic hypotension at inclusion but without urinary disorder at inclusion, with no delay since symptom onset and not receiving L-dopa, antihypotensive agents or antidepressants.

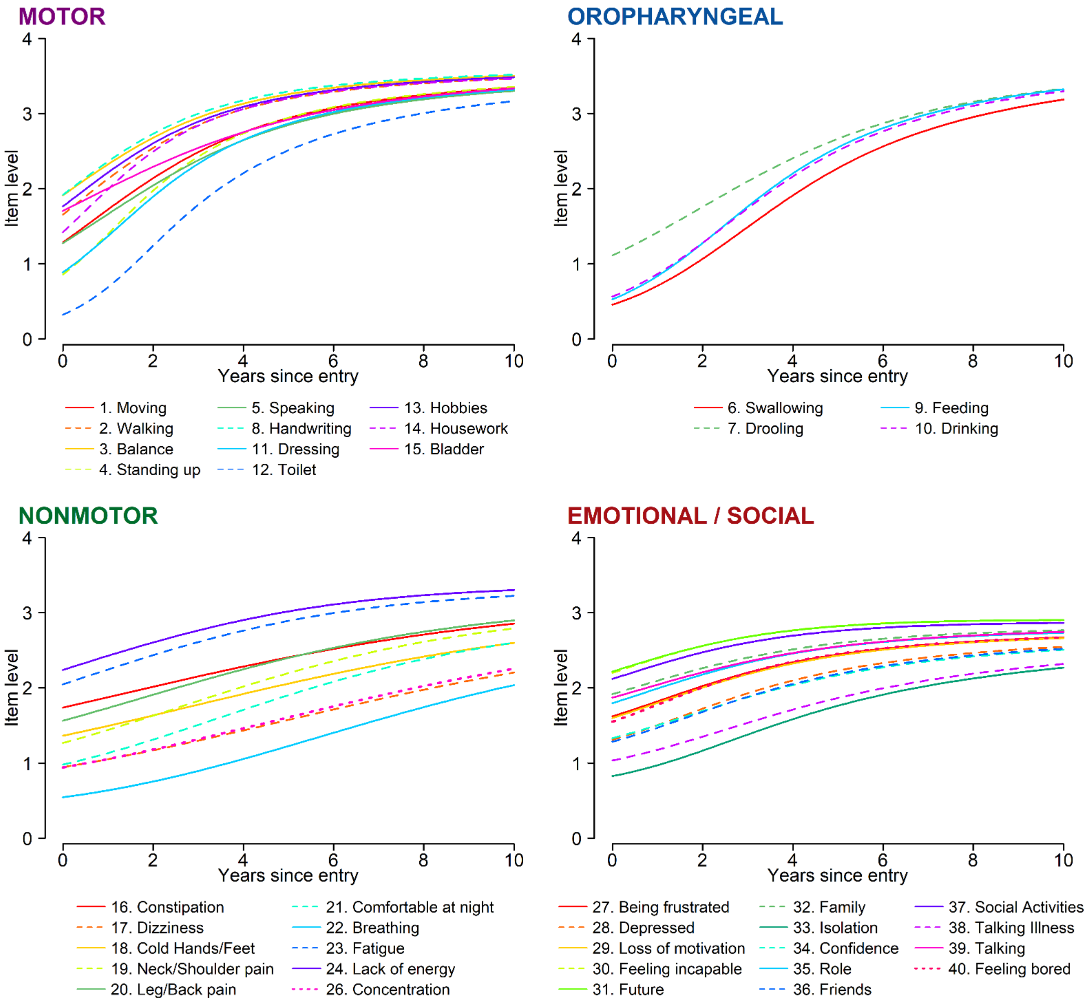

Appendix 1: Further details on the four steps constituting the MSA-QoL analysis strategy

Statistical analyses were carried out in R. A replication script is available at *https://github.com/TiphaineSAULNIER/4StepStrategy*.

1. Step 1: Identification of homogeneous MSA-QoL subscale dimensions

The different MSA-QoL dimensions measured by the scale were distinguished using the PROMIS methodology,[1] ensuring the validity of the three calibration assumptions of the items: unidimensionality, conditional independence, and increasing monotonicity. This method permitted us to successively evaluate these assumptions. However, the authors recommended some stepbacks to measure the impact of certain decisions and to ensure that the identified dimensions made clinical sense. The methods used did not handle repeated data, so we performed the first step on all follow-up data by neglecting the intrasubject correlation.

First, an explanatory factorial analysis (EFA) was performed on all items to identify the different phenomena measured by the questionnaire. The optimal number of dimensions was determined according to the scree plot of the successive eigenvalues and based on the greatest number of factors with successive eigenvalues greater than 1 or the Kaiser criterion.[2] This analysis was carried out using the function *fa.parallel()* from the R package *psych*. Then, each item was assigned to the dimension to which it most contributed, according to the polychoric correlation matrix. Afterwards, to confirm the result and to ensure the sufficient unidimensionality of the identified dimensions (i.e., all items from a dimension measure the same phenomenon), an EFA was performed for each dimension to control that the number of underlying factors was 1, and a confirmatory factorial analysis (CFA) was performed to evaluate the model fit based on PROMIS-recommended criteria thresholds: comparative fit index (CFI) > 0.95, Tucker Lewis index (TLI) > 0.95, root mean square error of approximation (RMSEA) < 0.06, and standardized root mean square residual (SRMR) < 0.08.[1,2] This analysis was performed using the function c*fa()* from the R package *lavaan*.

To ensure conditional independence (i.e., items from the same dimension do not carry redundant information), the residual correlation matrix between the CFA-fitted values and the observed values of the items for each dimension was computed. According to PROMIS, the assumption is not satisfied for a residual correlation greater than 0.2 between two items, and in this case, removing one item is recommended.

To ensure increasing monotonicity (i.e., higher levels of items always correspond to higher levels of QoL impairment), a nonparametric IRT model was performed for each dimension using the function *check.monotonicity()* from the R package *mokken*. For each item, it computes the probabilities of endorsing a higher level and predicts the item level to be compared to the increasing dimension sum scores (except for the considered item score) through plots. According to the authors, the item response curves should be increasing or at least constant.

At this stage, each homogenous subscale was identified and analysed separately in Steps 2 to 4.

2. Step 2: Description of MSA-QoL item trajectories over time and associated factors

The trajectory of each dimension continuum was modelled over time from the repeated item data using a joint item response theory (IRT) model adapted to ordinal repeated measures and time-to-event data.[3] The model, described in Figure S1, was simultaneously composed of a longitudinal submodel and a survival submodel, estimated by maximum likelihood in the R package JLPM[4] (https://github.com/VivianePhilipps/JLPM). The longitudinal submodel combined the following:

(i) a linear mixed structural model to describe the underlying dimension deterioration over time according to covariates and functions of time, with the fixed effects defining the mean dimension trajectory at the population level and individual correlated random effects capturing individual deviations, and

(ii) an item-specific cumulative probit measurement model to define the link between the underlying dimension and each item observation.

The survival submodel was a proportional hazard survival model adjusted using the underlying dimension dynamics as a linear predictor to account for the informative dropout induced by deaths. For further details, please refer to Saulnier et al.[3]

Saulnier T, *et al. J Neurol Neurosurg Psychiatry* 2024; 95:804–811. doi: 10.1136/jnnp-2023-332733

Figure S3: Joint IRT model structure for a latent dimension measured by K repeated items and the time of death.

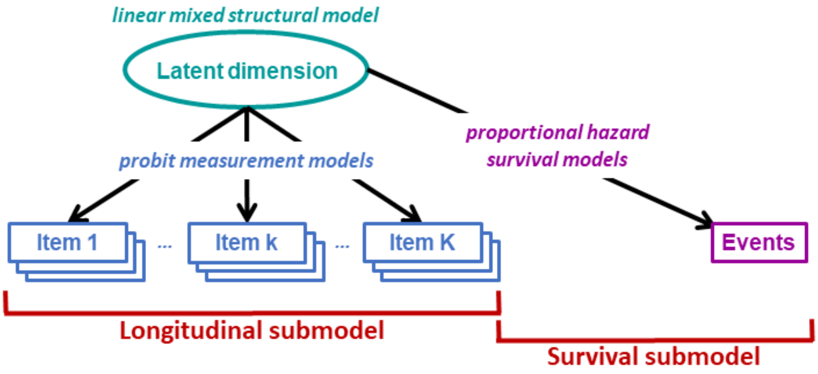

3. Step 3: Mapping item impairment hierarchy to disease stages

The disease stages were projected on a dimension continuum using a joint bivariate model to link the disease stages to the dimension total sum score. This was performed using the R package *JLPM*, which was also adapted for continuous markers by replacing the cumulative probit measurement model with linear and curvilinear measurement models (the curvilinear model involves a parameterized bijective link function approximated by splines).[3,4] Then, thresholds in the dimension continuum corresponding to each disease stage were deduced by predicting the dimension sum scores that corresponded to a change in disease stage and expressing them in the dimension process scale. As this part requires the computation of dimension sum scores, it does not handle missing data. To limit the number of excluded data, dimension sum scores were computed in proportion to the number of missing items, as long as there were less than 25% missing item values. This optimal threshold was chosen as a balance between a maximal number of observations and a minimal proportion of missing items.

4. Step 4: Listing the most informative items by disease stage

The contribution of each item was quantified by the percentage of the carried information at each stage. The information was defined by the Fisher information function (i.e., the second derivative of the item probability with respect to the underlying dimension), which was integrated over all the underlying dimension values corresponding to a specific stage (as determined in Step 3) to obtain the item- and stage-specific information. The total information of a dimension at a specific stage was the sum of all item- and stage-specific

information so that the percentage of total information carried by an item at a disease stage was easily deduced.

References

1. Reeve BB, Hays RD, Bjorner JB, et al. Psychometric evaluation and calibration of health-related quality of life item banks: plans for the Patient-Reported Outcomes Measurement Information System (PROMIS). *Med Care*. 2007;45(5 Suppl 1):S22-31. doi:10.1097/01.mlr.0000250483.85507.04

2. Lee CT, Zhang G, Edwards MC. Ordinary Least Squares Estimation of Parameters in Exploratory Factor Analysis With Ordinal Data. *Multivariate Behav Res*. 2012;47(2):314-339. doi:10.1080/00273171.2012.658340

3. Saulnier T, Philipps V, Meissner WG, et al. Joint models for the longitudinal analysis of measurement scales in the presence of informative dropout. *Methods*. 2022;203:142-151. doi:10.1016/j.ymeth.2022.03.003

4. Philipps V, Saulnier T, Proust-Lima C. *JLPM: Joint Latent Process Models*.; 2022. Accessed October 28, 2022. https://CRAN.R-project.org/package=JLPM

Table S1: Ranking of MSA-QoL items per dimension for the 5 UMSARS-IV stages according to the item-specific Fisher information carried

	Stage I			Stage II			Stage III			Stage IV			Stage V		
Rank	Item	Info %	Cum Info %	Item	Info %	Cum Info %	Item	Info %	Cum Info %	Item	Info %	Cum Info %	Item	Info %	Cum Info %
**MOTOR dimension**															
1	2. Walk	18.0	18.0	4	17.9	17.9	4	17.8	17.8	12	17.1	17.1	5	39.1	39.1
2	3. Balance	15.4	33.4	14	13.2	31.1	12	14.1	31.9	4	13.7	30.8	15	32.9	71.9
3	14. Housework	11.9	45.3	11	12.4	43.5	11	12.6	44.6	11	13.2	44.1	1	18.9	90.8
4	8. Handwriting	11.2	56.5	2	12.3	55.8	2	11.4	55.9	2	10.9	55.0	12	3.4	94.2
5	1. Move	9.5	66.0	12	9.3	65.0	14	11.3	67.2	1	10.8	65.7	11	1.7	95.9
6	13. Hobbies	8.7	74.6	1	8.3	73.3	1	8.0	75.2	5	8.1	73.8	13	1.6	97.4
7	5. Speak	8.3	82.9	3	7.6	81.0	3	7.1	82.3	3	7.2	81.0	3	1.4	98.8
8	11. Dress	6.5	89.4	8	5.9	86.9	5	5.7	88.0	14	6.6	87.6	8	0.7	99.5
9	4. Stand up	5.7	95.1	5	5.7	92.7	8	5.2	93.2	13	4.9	92.5	2	0.4	99.9
10	15. Bladder	4.7	99.8	13	5.4	98.1	13	4.9	98.1	8	4.7	97.3	4	0.1	100.0
11	12. Toilet	0.2	100.0	15	1.9	100.0	15	1.9	100.0	15	2.7	100.0	14	0.0	100.0
**OROPHARYNGEAL dimension**															
1	7. Saliva	58.4	58.4	9	33.9	33.9	9	35.4	35.4	9	35.0	35.0	6	36.6	36.6
2	10. Drink	19.7	78.1	10	31.7	65.6	10	31.0	66.5	10	30.8	65.8	10	28.0	64.6
3	9. Feed	11.3	89.4	6	23.2	88.8	6	24.1	90.6	6	25.2	91.0	9	22.5	87.1
4	6. Swallow	10.6	100.0	7	11.2	100.0	7	9.4	100.0	7	9.0	100.0	7	12.9	100.0
**NONMOTOR dimension**															
1	24. Energy	26.8	26.8	23	18.5	18.5	23	17.9	17.9	23	17.2	17.2	21	15.2	15.2
2	23. Tired	24.5	51.2	24	18.3	36.8	24	17.6	35.4	24	16.8	33.9	22	13.9	29.1
3	20. Legs/Back pain	10.9	62.1	19	13.0	49.8	21	13.4	48.9	21	13.8	47.8	19	12.3	41.4
4	19. Neck/Shoulders pain	9.1	71.3	21	13.0	62.8	19	13.2	62.0	19	13.3	61.0	26	11.4	52.8
5	16. Constipation	8.2	79.5	20	10.3	73.1	20	10.2	72.3	20	10.2	71.2	20	9.7	62.5
6	21. Comfortable	6.0	85.5	22	6.5	79.6	22	7.1	79.4	22	7.7	79.0	17	8.8	71.3
7	18. Cold Hands/Feet	5.3	90.9	26	5.9	85.5	26	6.1	85.4	26	6.3	85.2	23	7.9	79.2
8	26. Concentration	4.3	95.2	16	5.2	90.7	16	5.2	90.6	16	5.1	90.3	18	7.8	86.9
9	17. Dizziness	3.3	98.5	18	5.0	95.7	18	5.0	95.6	18	5.1	95.4	24	6.7	93.6
10	22. Breath	1.5	100.0	17	4.3	100.0	17	4.4	100.0	17	4.6	100.0	16	6.4	100.0
**EMOTIONAL/SOCIAL dimension**															
1	30. Incapable	13.5	13.5	40	13.1	13.1	40	13.2	13.2	40	12.9	12.9	28	11.7	11.7
2	31. Future	11.6	25.1	29	10.2	23.3	28	10.3	23.5	28	10.5	23.5	33	11.0	22.7
3	29. Motivation	10.2	35.3	28	10.1	33.4	29	10.1	33.6	29	10.1	33.6	34	10.3	33.1
4	35. Role	9.3	44.6	30	8.3	41.7	27	8.0	41.6	27	8.0	41.5	29	9.8	42.8
5	40. Boring	8.1	52.7	27	8.1	49.7	30	7.9	49.5	30	7.5	49.1	36	8.9	51.7
6	37. Social Activities	7.9	60.6	35	7.6	57.4	35	7.5	57.0	35	7.4	56.5	40	7.5	59.2
7	32. Family	7.7	68.3	31	6.9	64.3	36	6.9	63.9	36	7.2	63.6	27	6.9	66.0
8	27. Frustration	7.4	75.7	36	6.6	70.9	31	6.6	70.5	34	6.7	70.3	38	6.8	72.8
9	39. Talk	6.3	82.0	34	6.4	77.4	34	6.5	77.1	31	6.3	76.7	35	6.7	79.5
10	28. Depression	6.1	88.1	32	5.3	82.7	33	5.4	82.5	33	5.9	82.6	32	5.5	85.0
11	34. Confidence	5.4	93.5	37	5.2	87.9	32	5.2	87.7	32	5.2	87.8	39	4.4	89.4
12	36. Friends	3.6	97.1	33	4.9	92.8	37	4.9	92.7	37	4.7	92.5	31	4.0	93.3
13	38. Talk Illness	1.5	98.6	39	4.2	97.0	39	4.1	96.8	39	4.1	96.6	30	3.9	97.2
14	33. Isolation	1.4	100.0	38	3.0	100.0	38	3.2	100.0	38	3.4	100.0	37	2.8	100.0

Info % percentage of Fisher information carried by the item,
Cum Info % cumulative percentage of Fisher information carried by the item and the most informative ones.
